# Efficacy and safety of CyberKnife radiosurgery in elderly patients with brain metastases: a retrospective clinical evaluation

**DOI:** 10.1186/s13014-020-01655-8

**Published:** 2020-09-29

**Authors:** Gueliz Acker, Seyed-Morteza Hashemi, Josch Fuellhase, Anne Kluge, Alfredo Conti, Markus Kufeld, Anita Kreimeier, Franziska Loebel, Melina Kord, Diana Sladek, Carmen Stromberger, Volker Budach, Peter Vajkoczy, Carolin Senger

**Affiliations:** 1 Department of Neurosurgery, Charité Universitätsmedizin Berlin, corporate member of Freie Universität Berlin, Humboldt-Universität zu Berlin, and Berlin Institute of Health, Charitéplatz 1, 10117 Berlin, Germany; 2 Berlin Institute of Health (BIH), Anna-Louisa-Karsch-Str. 2, 10178 Berlin, Germany; 3 Charité CyberKnife Center, Augustenburger Platz 1, 13353 Berlin, Germany; 4 Department of Radiation Oncology, Charité Universitätsmedizin Berlin, corporate member of Freie Universität Berlin, Humboldt-Universität zu Berlin, and Berlin Institute of Health, Augustenburger Platz 1, 13353 Berlin, Germany; 5 Department of Neurosurgery, Biomedical and Neuromotor sciences, Alma Mater Studiorum University of Bologna, Bologna, Italy

**Keywords:** CyberKnife, Stereotactic radiosurgery, Elderly patients, Brain metastases

## Abstract

**Background:**

Stereotactic radiosurgery (SRS) has been increasingly applied for up to 10 brain metastases instead of whole brain radiation therapy (WBRT) to achieve local tumor control while reducing neurotoxicity. Furthermore, brain-metastasis incidence is rising due to the increasing survival of patients with cancer. Our aim was to analyze the efficacy and safety of CyberKnife (CK) radiosurgery for elderly patients.

**Methods:**

We retrospectively identified all patients with brain metastases ≥ 65 years old treated with CK-SRS at our institution since 2011 and analyzed data of primary diseases, multimodality treatments, and local therapy effect based on imaging follow-up and treatment safety. Kaplan–Meier analysis for local progression-free interval and overall survival were performed.

**Results:**

We identified 97 patients (233 lesions) fulfilling the criteria at the first CK-SRS. The mean age was 73.2 ± 5.8 (range: 65.0–87.0) years. Overall, 13.4% of the patients were > 80 years old. The three most frequent primary cancers were lung (40.2%), kidney (22.7%), and malignant melanoma (15.5%). In 38.5% (47/122 treatments) multiple brain metastases were treated with the CK-SRS, with up to eight lesions in one session. The median planning target volume (PTV) was 1.05 (range: 0.01–19.80) cm^3^. A single fraction was applied in 92.3% of the lesions with a median prescription dose of 19 (range: 12–21) Gy. The estimated overall survivals at 3-, 6-, and 12 months after SRS were 79, 55, and 23%, respectively. The estimated local tumor progression-free intervals at 6-, 12-, 24-, 36-, and 72 months after SRS were 99.2, 89.0, 67.2, 64.6, and 64.6%, respectively. Older age and female sex were predictive factors of local progression. The Karnofsky performance score remained stable in 97.9% of the patients; only one patient developed a neurological deficit after SRS of a cerebellar lesion (ataxia, CTCAE Grade 2).

**Conclusions:**

SRS is a safe and efficient option for the treatment of elderly patients with brain metastases with good local control rates without the side effects of WBRT. Older age and female sex seem to be predictive factors of local progression. Prospective studies are warranted to clarify the role of SRS treatment for elderly patients.

## Background

Stereotactic radiosurgery (SRS), in place of whole brain radiation therapy (WBRT), is the standard for patients with 1–3 metastases and an effective treatment for patients with up to 10 lesions to reduce neurotoxicity and preserve quality of life [1–5]. Brain metastases incidence has been increasing and will continue to rise due to the improvement of systemic therapies [1, 2, 6]. In the current era of immunotherapy or other targeted therapies, overall survival (OS) has increased; thus, the demand for less toxic alternatives than WBRT and the need for retreatment with radiosurgery for local progression or for new cerebral lesions is on the rise [2, 7, 8]. It has been postulated that in the near future, 70% of new cancer diagnoses per year will be reported among the elderly [9, 10]. Consequently, clinicians are facing a larger population of aging patients.

The management of the elderly remains an issue of debate, as they comprise an inhomogeneous patient population with diverse comorbidities and different levels of fitness. Age and the Karnofsky performance score (KPS) have been used to roughly categorize the patients, as proposed by the recursive partitioning analysis (RPA) for prognostic factors by the Radiation Therapy Oncology Group, where patients ≥ 65 years old were classified as patients at medium risk with RPA class II [11]. For patients ≥ 70 years who received GammaKnife SRS of their brain metastases, Park et al. identified the graded prognostic assessment (GPA) classification as a strong prognostic factor for survival [12]. Nevertheless, elderly patients are generally underrepresented in randomized clinical trials, and thus clinical practice is based on small patient series and personal experience and assessment of the treating physician.

Importantly, SRS may represent the treatment of choice considering the risk of cognitive decline in the elderly after WBRT [13, 14]. However, few reports have assessed the feasibility and efficacy of SRS in the elderly using conventional linear accelerator (LINAC) or GammaKnife-based SRS, and to our knowledge, none have done so using a frameless technique with the CyberKnife (CK, Accuray Inc., Sunnyvale, CA), an image-guided robotic linear accelerator system [15–18]. Thus, our aim in this study was to characterize our elderly patient cohort with brain metastases and to analyze the efficacy and safety of CK-SRS in this population.

## Methods

### Study design

This retrospective analysis of patient data was approved by the local ethics committee (EA1/233/18). We identified all patients with brain metastases who were ≥ 65 years old at the time of the first CK-SRS and who were treated at our center between July 2011 and August 2018. We collected data on patient characteristics regarding the primary disease, treatment modalities, clinical outcome, local and overall tumor control, and acute and long-term treatment morbidity. The KPS score prior and after the treatment was used to classify the clinical status and course of the patients. New neurological deficits were documented. To evaluate quality of life and neurocognitive effects all answered EORTC QLQ-C30 and QLQ-BN20 questionnaires were additionally analyzed. The Common Terminology Criteria for Adverse Events 5.0 (CTCAE) were used to report adverse effects [19]. We also analyzed radiometric parameters such as prescription dose, fractionation scheme and planning target volume (PTV). The graded prognostic assessment (GPA) score considering the age, KPS, the presence of extracranial metastases, and the number of brain metastases was calculated. For patients ≥ 65 years of age, a score of 2.5–3.0 (Class 2) correlates with the best prognosis, and a score of 0–1.0 points (Class 4) correlates with the worst prognosis [20].

### CyberKnife treatment

All patients included in the study were referred for CK-SRS for a limited number of brain metastases. Resection cavities were excluded from this analysis. The indication for CK treatment was decided by a multidisciplinary neuro-oncology board team including a radiation oncologist and a neurosurgeon. A thermoplastic mask was individually produced for each patient for treatment immobilization before contrast enhanced high-resolution thin-slice (0.75 mm) computed tomography (CT). This reference CT was co-registered to T1-weighted magnetic resonance images (MRI: magnetization-prepared rapid acquisition with gradient echo using gadolinium-based contrast agents, 1.0 mm slice thickness) using MultiPlan (Accuray Inc.).

The planning process with the prescription of dose, fractionation scheme, target definition, and dose optimization was executed by a team comprising a radiation oncologist, a neurosurgeon, and a radiation physicist. The gross tumor volume (GTV) was defined as the tumor volume based on contrast-enhanced CT and MRI. PTV margins of 0–1 mm were chosen by the physician under consideration of MRI quality and actuality, a size progression between MRI and planning CT or if no contrast agent could be administered during planning CT.

Different dose regimens were applied depending on the closeness to organs at risk (OARs; such as the optic nerves, chiasm, and brainstem), the size and previous treatments. In general, SRS for brain metastases with a diameter ≤ 2.0 cm is performed in single fraction and  > 2.0 cm in 3 fractions. If a brain metastasis is eloquently located (e.g. in the brainstem or along the optic pathway), either a reduction of the single fraction dose or hypofractionation is performed, depending on how the dose constraints are met. In case of a local recurrence after a single dose SRS, the CyberKnife reirradiation is preferably fractionated. For metastases ≤ 2 cm, the most commonly administered dose was 20–21 Gy in single-fraction SRS; for larger brain metastases, multisession SRS with three fractions of 8–9 Gy and a total dose of 24 to 27 Gy was used. The doses were routinely prescribed to the 70% isodose line covering the PTV. Dose distributions were calculated with the Ray-tracing algorithm. Dose constraints to OARs for single fraction CK-SRS were as follows: ≤ 0.2 cm^3^ of the optic pathway could receive 8.0 Gy with a maximum point dose of 10.0 Gy in ≤ 0.035 cm^3^, and ≤ 0.35/≤ 1.2 cm^3^ of the brainstem (medulla) could receive 10.0/7.0 Gy with a maximum point dose of 14.0 Gy in ≤ 0.035 cm^3^ [21]. The eyes were generally not directly irradiated. The biological equivalent dose for 2 Gy per fraction was calculated according to the LQ-model assuming an α/β-ratio of 2 Gy for normal brain tissue (EQD2_2_) and 10 Gy for tumoral tissue (EQD2_10_). The calculated EQD2_10_ encompassing the PTV was 50.0–54.3 Gy for a single fraction, 36.0–42.8 Gy for three-fraction treatment and 31.3 Gy for five-fraction treatment.

SRS was performed with a non-isocentric treatment technique, tracking the patient’s skull approximately every 60 s with x-rays for accurate beam delivery. One session lasted between 30 and 120 min. Patients routinely received a single dose of 4 mg dexamethasone after the treatment to prevent adverse effects due to post-radiosurgical tumoral or normal tissue swelling.

### Follow-up

Every 3 months, a clinical evaluation and radiological imaging using contrast-enhanced MRI were evaluated as a follow-up assessment. The latest available follow-up was included in this analysis. The KPS and new neurological deficits were separately documented prior to the treatment and at the last follow-up. The MRI scans were analyzed by the responsible physician to assess the response to treatment. We first examined the local control in the area of the PTV. The treatment response of each lesion was assessed by the Response Assessment in Neuro-Oncology Brain Metastases (RANO-BM) [22]. Local tumor control was achieved if the treated brain metastases showed a complete response (disappearance of the lesion), partial response (at least a 30% decrease in the longest dimension), or stable disease without significant change in size. Local progression of the disease was assumed if a lesion increased by at least 20% in diameter, and distant failure was defined as new metastases diagnosed based on the follow-up MRI scans. OS was calculated from the first CK-SRS until the analysis for this study (07/19). The Berlin–Brandenburg tumor registry was used to verify the surveillance.

### Statistical analysis

Overall survival (OS) and progression-free survival were investigated using Kaplan–Meier analysis. Progression-free survivals for 6, 12, 24, 36, 48, and 60 months were calculated. Group comparisons were carried out using log-rank tests. To assess the risk factors possibly associated with earlier local recurrence of brain metastases, univariate and multivariate analyses were performed using Cox regression analysis; parameters with *p* < 0.05 in the univariate analyses were entered into the multivariate analysis. The data are presented as median and range. IBM SPSS Statistics (Version 25.0. Armonk, NY: IBM Corp.) was used, and a *p* ≤ 0.05 was regarded as significant.

## Results

### Patient characteristics

We analyzed the data of 233 treated lesions of 97 patients with brain metastases. Demographic and clinical characteristics are summarized in Table 1. Overall, 54.6% of the patients were between 70 and 80 years old and 13.4% were > 80 years; to attain a fair distribution of the entire cohort, we categorized the patients in three groups: < 70 (37.3%), 70 to 75 (32.2%), > 75 (30.5%) years old. More than half of the patients were male (58.8%). The three most frequent primary tumor types were lung (40.2%), kidney (22.7%), and malignant melanoma (15.5%; Table 1). A total of 14 patients had undergone WBRT, 10 patients before and 3 patients after CK-SRS, while in one case the time point was not documented (Table 1). In the patient cohort, 29.9% (*n* = 29) of all patients had undergone a tumor resection prior to CK-SRS for another lesion (Table 1). Additional systemic treatment with chemotherapy and/or immunotherapy was performed in 82.5% (*n* = 80) of the patients, while in 72.2% of the patients a chemo- or antihormonal therapy was documented. There was no relevant difference observed between the age groups (Table 2).

**Table 1:** Summary of patient characteristics

	Overall cohort
Number	97
Sex	
Male	58.8%
Female	41.2%
Age at diagnosis in years	
Mean ± SD	70.1 ± 8.3
Age at 1. CK SRS in years	
Mean ± SD	73.3 ± 5.8
Years from diagnosis to CK SRS	
Mean ± SD	3.2 ± 5.0
Number of lesions per treatment	
1	53.0%
2–4	39.9%
5–7	6.6%
8	0.5%
Number of treatments per patient	
1	79.5%
2	16.4%
> 2	4.1%
Pathologies	
Lung cancer	40.2%
Renal cell carcinoma	22.7%
Malignant melanoma	15.5%
Breast cancer	10.3%
Gastrointestinal tract	3.1%
Others	3.1%
Pharynx	2.1%
Cancer of unknown pathology	2.1%
Urothelial carcinoma	1.0%
Whole brain irradiation treatment (% of patients)	
Without	83 (85.6%)
Prior to CK-SRS	10 (10.3%)
After CK-SRS	3 (3.1%)
GPA Class	
2	5 (5.2%)
3	58 (59.8%)
4	34 (35.1%)
Extracranial metastases at diagnosis	
Yes	72 (74.2%)
No	25 (25.8%)

*CK* CyberKnife, *SRS* Stereotactic radiosurgery, *GPA* Graded prognostic assessment

**Table 2:** Patients with systemic therapies as % of all treated patients (*n* = 97)

	Chemotherapy/anti-hormonal therapy	Immuno−/targeted therapy	Chemo- and immunotherapy	Systemic therapy (Chemo-and/or immunotherapy)
All patients	72.2	46.4	36.1	82.5
Age < 70	27.8	15.5	13.4	29.9
Age 70–75	26.8	13.4	11.3	28.9
Age > 75	17.5	17.5	11.3	23.7
Unknown	17.5	15.5	12.4	12.4

### CK-SRS treatment characteristics

In 38.5% (47/122 treatments) of treatments, multiple brain metastases were treated with CK-SRS, with up to eight lesions in one session. In total, 14.4% of the patients (14 of 97) were treated repeatedly (up to four times) with CK-SRS. The median PTV for the lesions was 1.05 cm^3^ (range: 0.01 cm^3^–19.80 cm^3^). A single fraction was used in 92.3% of the lesions with a median prescription dose (PD) of 19 Gy (range: 12–21 Gy) and a PTV of 0.93 cm^3^ (range: 0.01 cm^3^–11.10 cm^3^). The median PTV for the lesions that received hypofractioned therapy in three fractions was 3.74 cm^3^ (range: 0.82 cm^3^–19.80 cm^3^) with a median PD of 27 Gy (range: 21–27 Gy). The PTV for the only lesion treated by 25 Gy in 5 fractions was 13.9 cm^3^.

### Overall survival and tumor control

**Fig. 1:**
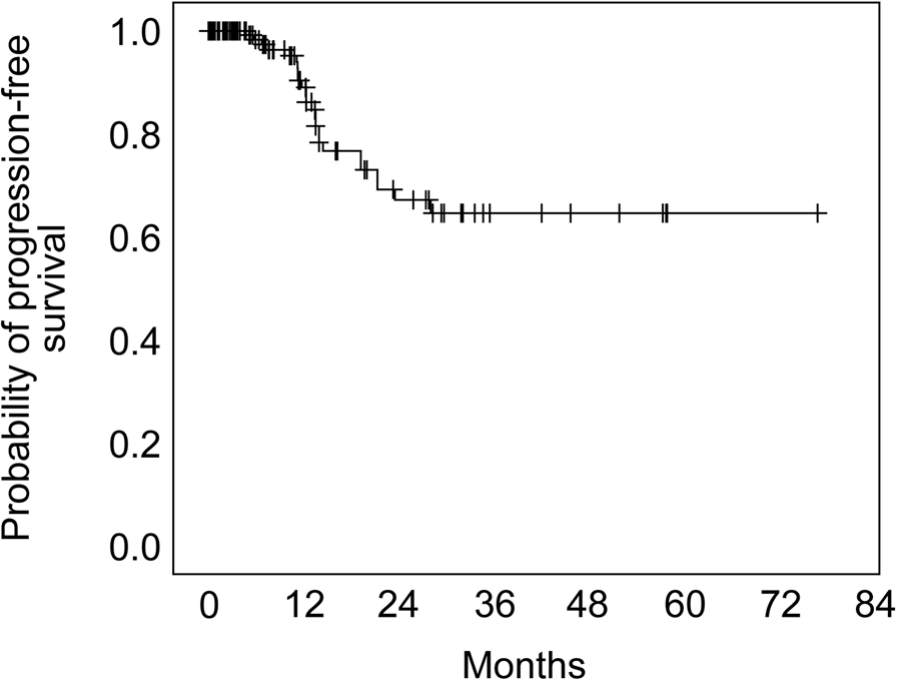
The overall estimated local tumor progression-free interval. Number of lesions at risk were 233 (0 months), 66 (12 months), 33 (24 months), 7 (36 months), 1 (60 months)

**Fig. 2:**
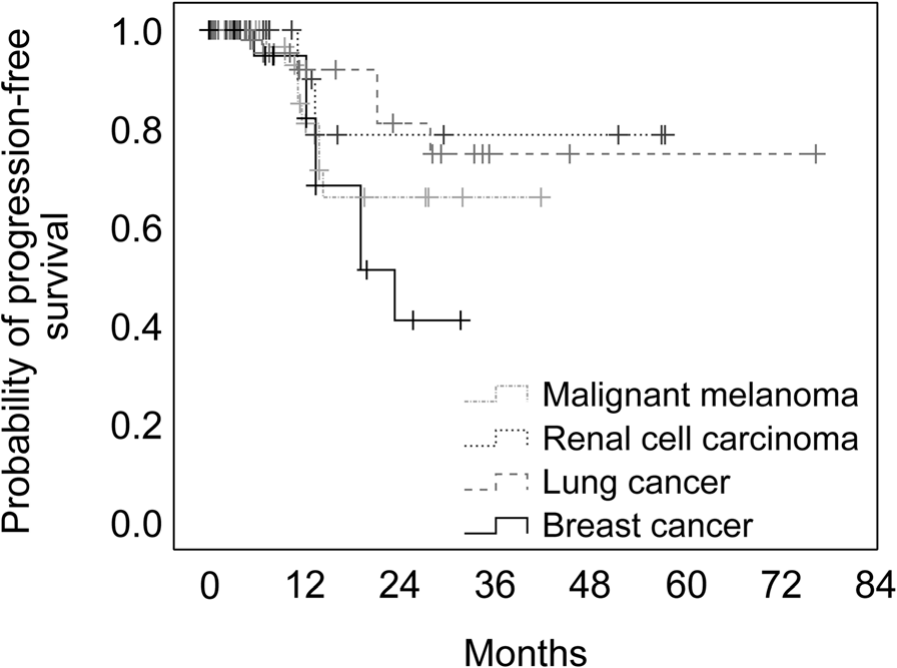
The overall estimated local tumor progression-free interval divided in the most frequent pathologies

**Fig. 3:**
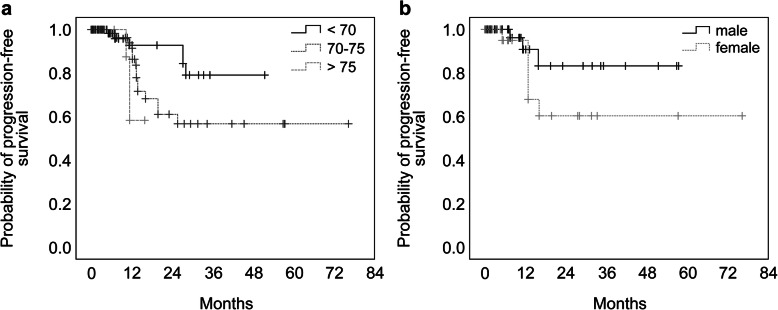
The overall estimated local tumor progression-free interval for different ages and sex. a, three groups divided by age < 70, 70 to 75, > 75 years. A significant difference was only seen between patients < 70 and > 75 years old (log rank test, *p* = 0.028). b, Furthermore, regarding the overall estimated local tumor progression-free interval of male and female patients, female patients had worse local control compared to male patients (log rank test, *p* = 0.003)

The estimated OS rates at 3-, 6-, and 12-months after SRS were 79, 55, and 23%, respectively. At least one follow-up assessment with sufficient imaging material was present in 77.3% of the patients and in 80.0% of the lesions with a median follow-up period of 7.6 months (range: 0.3–76.3 months). The estimated local tumor progression-free interval fractions at 6-, 12-, 24-, 36-, and 72 months were 99.2, 89, 67.2, 64.6, and 64.6%, respectively (Fig. 1). There were differences amongst the different tumor origins (Fig. 2). Malignant melanoma metastases achieved the best local control rates, although without significant differences in comparison to the other pathologies. The only significant difference was detected between lung and breast cancer metastases, with worse local control for breast tumors (Fig. 2; log rank test, *p* = 0.034). A significant difference was seen between patients < 70 and > 75 years old (Fig. 3a; log rank test, *p* = 0.028). Furthermore, we compared local progression between sexes, and found that local control was worse for female patients (Fig. 3b; log rank test, *p* = 0.003). In this regard, we analyzed the proportion of radioresistant tumors amongst both sexes. A total of 20.0% of female and 24.6% of the male patients had a renal cell carcinoma, malignant melanoma was diagnosed in 12.5% of females and 19.3% of males. The radiation therapy resistant histologies like malignant melanoma and renal cell carcinoma were balanced between the sexes. Of the 9 female patients with breast cancer, only one was diagnosed with triple negative.

**Fig. 4:**
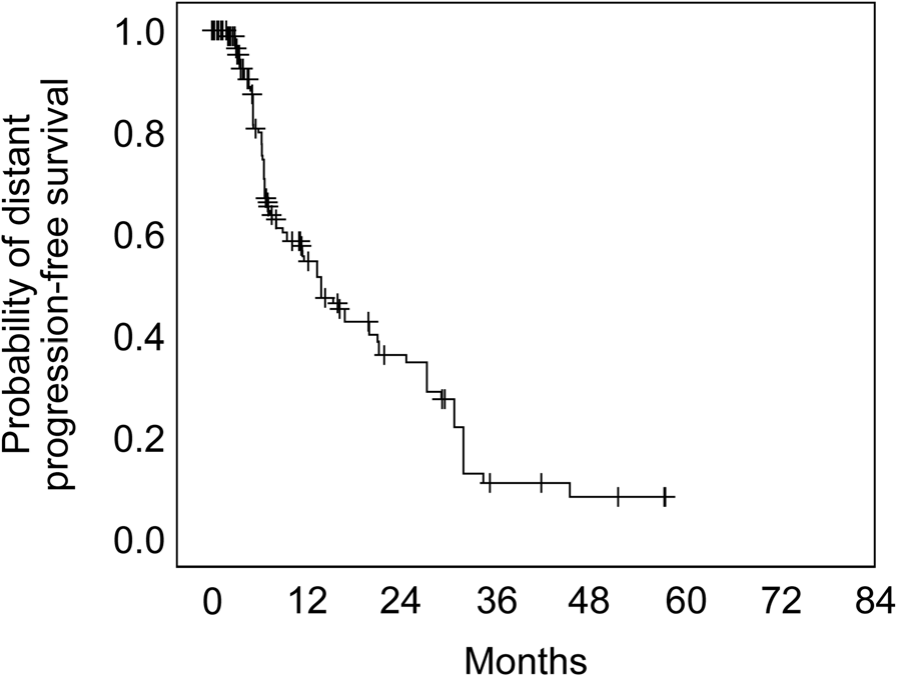
The overall estimated distant tumor progression-free interval. Number of lesions at risk were 233 (0 months), 54 (12 months), 25 (24 months), 5 (36 months), 0 (60 months)

Categorization of the local response could be performed in 70.0% of the lesions with a minimum follow-up period of 3 months (median: 10.3 months with a range of 3.0–76.3 months; Table 3). Nearly half of the lesions (46.3%) achieved partial remission, whereas only 14.8% of the lesions showed local progression. Complete remission was accomplished in 22.2% of the lesions. The estimated distant tumor progression-free interval fractions at 6-, 12-, 24-, 36-, and 72 months were 80.0, 54.6, 36.2, 11.0, and 8.3%, respectively (Fig. 4).

**Table 3:** Summary of different local response categories in a total of 162 lesions with a minimum follow-up of 3 months (median: 10.3 months with a range of 3.0–76.3 months)

	Number of lesions	%
Complete remission	36	22.2
Partial remission	75	46.3
Stable disease	27	16.7
Progressive disease	24	14.8

### Factors affecting local control

In the univariate cox proportional hazards regression analyses, older age, female sex, and larger PTV were the risk factors that reached significance (Table 4). In the multivariate analysis, only older age and female sex remained significant risk factors for local recurrence, while PTV reached *p* = 0.051, remaining slightly above the threshold of significance.

**Table 4:** Univariate and multivariate Cox regression analyses for factors affecting the time to local tumor control

	Univariate Analyses			Multivariate Analysis		
	HR	95% CI	***p***-value	HR	95% CI	***p***-value
Age	1.136	1.048–1.231	**0.002**	1.135	1.028–1.258	**0.012**
Sex (male)	0.247	0.092–0.662	**0.005**	0.270	0.100–0.730	**0.010**
PTV	1.186	1.051–1.339	**0.006**	1.128	0.996–1.277	0.058
Prescribed dose*	0.732	0.498–1.074	0.111			
Primary tumor	1.025	0.902–1.166	0.703			
GPA class	1.266	0.633–2.530	0.505			

*HR* Hazard ratio, *CI* Confidence interval, *PTV* Planning target volume, *GPA* Graded prognostic assessment. *for the prescribed dose only single fractions were included. bold text: *p* ≤ 0.05 was regarded as significant

### Morbidity, mortality and quality of life

**Fig. 5:**
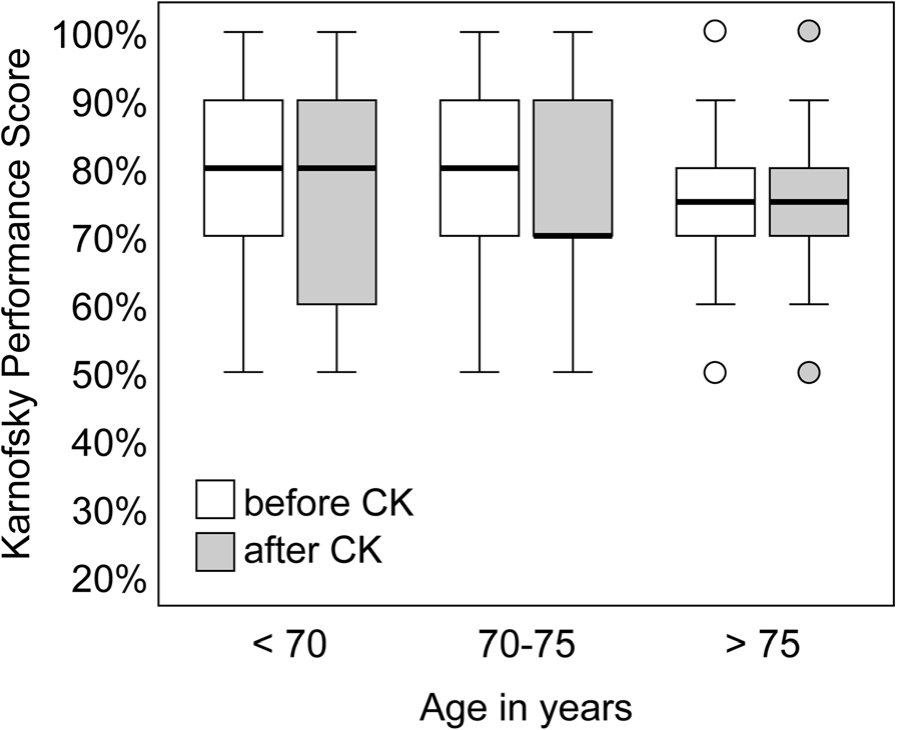
A boxplot diagram for the Karnofsky Performance Score (KPS) before and after treatment. Three groups divided by age (< 70, 70 to 75, > 75 years) showing an overall stable KPS after treatment. The boxes represent the interquartile range, the thicker line inside the boxes the median, and the whiskers indicate the range from minimum to maximum, excluding outliers (circles)

No severe (>CTCAE Grade 2) acute complications occurred. Overall, KPS remained stable in 97.9% of the patients, whilst only 1.0% deteriorated by ≥ 10 points within the follow-up period. Patients > 75 years old also tolerated the treatment well without major deterioration (Fig. 5). Only one patient (1.0%) developed a new neurological deficit after SRS of a cerebellar lesion (ataxia, CTCAE Grade 2). White matter changes such as edema and/or necrosis as asymptomatic radiation-induced imaging signs occurred in four patients (4.0%), while intracranial hemorrhage developed in only one patient with melanoma in four of five lesions without symptoms due to minor bleeding. Overall, the rate of intracranial hemorrhage for the patients with melanoma was patient-based 6.6% (1 in 15 patients) and lesion-based 14.8% (4 in 27 lesions).

Of all 97 patients, two EORTC questionnaires (QLQ-C30, QLQ-BN20) were available for 60–68 patients, at first irradiation and 17–20 patients answered a follow-up questionnaire, respectively. The median follow-up time for the last latest questionnaire was 11 months (range: 3–35 months). Mean scores of the subscales are listed in Table 5. Physical and social functioning in EORTC QLQ-C30 deteriorated after SRS, while drowsiness and motor dysfunction were evaluated worse in EORTC QLQ-BN20.

**Table 5:** Average scores of the two EORTC quality of life scales answered before the first stereotactic radiosurgery (SRS) and the last available questionnaire per patient. *Subscales of EORTC QLQ-C30: higher score is better. ^#^For QLQ-BN20 subscales, a higher score is worse. n = existing evaluable answers

	Before SRS	Latest follow-up
EORTC QLQ – C30*	*n* = 63–68	*n* = 20
Global health status/Quality of life	48.4	46.0
Physical functioning**	63.3	55.8
Cognitive functioning	65.4	63.3
Role functioning	55.5	49.2
Emotional functioning	60.1	59.6
Social functioning**	58.5	45.0
EORTC QLQ – BN20^#^	*n* = 60–62	*n* = 17
Visual disorder	30.1	49.0
Motor dysfunction**	48.1	60.8
Communication deficit	21.0	27.5
Headaches	27.3	23.5
Seizures	4.4	2.0
Drowsiness**	42.1	52.9
Weakness of legs	35.2	37.3

** *p* < 0.05 (pairwise Wilcoxon signed-rank test for the comparison before and after SRS)

## Discussion

Our study evaluated for the first time the efficacy and safety of image-guided frameless CK-SRS in a large cohort of elderly patients. Our study supports the recommendation of SRS for this population in light of the reasonable local control rates with very rare complications.

SRS provided advantages concerning the risk of neurocognitive decline in an elderly population after radiation therapy [13, 14]. Besides, the convenience of a short treatment in an outpatient setting should be considered as an advantage. However, the accumulated experience with SRS for this specific patient population is based on few reports using LINAC or GammaKnife-based SRS [15–18]. As the OS after cancer diagnosis is on the rise due to new therapeutic regimens, the role of radiosurgery will become more important for the treatment of the elderly [2, 7–10].

In our series, the median follow-up time was 7.6 months with a wide range (0.3–76.3 months) due to the different survival times of the patients. However, the portion of the patients with a follow-up period more than 2 years was low with 14.4%. The estimated local control rates at 6-, 12-, 24-, 36-, and 72 months were 99.2, 89.0, 67.2, 64.6, and 64.6%, respectively. Nearly half of the lesions achieved partial remission, whereas only 14.8% showed local progression, defined as 20% tumor growth. The most recent report using a LINAC-SRS system analyzed 110 cerebral metastases in 40 patients [18]. In that series, by Gregucci et al., the complete and partial remission rates were comparable to those in our series (complete remission: 10.9% vs. 22.2%; partial remission: 46.4% vs. 46.3%), while the progressive disease rate was lower in their series (1.0% vs. 14.8%) [17]. This discrepancy is most probably due to the different definition of progression; they defined progression as 50% tumor growth [18] compared to our series where it was defined as 20%. Noel et al. reported on approximately 227 metastases in 117 elderly patients also using LINAC-SRS with a local control rate of 91% at 12 months, similar to our series [15]. Minniti et al. also reported the results of a series of 102 elderly patients with 183 metastases treated with LINAC-SRS who attained 84% local control at 12 months (17). Kim et al. analyzed 74 metastases in 44 elderly patients treated with GammaKnife, where local progression was observed in 12% of the patients [16]. Chen et al. compared 37 elderly patients treated with CyberKnife or LINAC-SRS with 82 patients treated by WBRT focusing on toxicity [23]. The authors concluded that WBRT was associated with greater toxicity compared to SRS in elderly and very elderly patients with brain metastases [23]. Gregucci et al. also reported no severe adverse effects worse than grade 2 after SRS [18]. Mininiti et al. reported a neurological complication rate of 13% [17]. In our series, KPS deteriorated by ≥ 10 points only in 1% of the patients, while neurological complications occurred only in 1% of patients. Overall, 14 patients underwent WBRT, which apparently did not cause more significant complications than SRS alone. Evidence regarding the safety of SRS after WBRT was recently reported by Lohkamp et al. [24], and was confirmed by our study findings where even fewer complications were observed.

Although, our study does not provide a head to head comparison of SRS to WBRT, we have to highlight the limitation of SRS in regard to the lack of distant control with our estimated distant progression-free rates. The prospective randomized trial by Brown et al. [5, 25] reported a significantly shorter time to intracranial failure for SRS alone compared with SRS plus WBRT (hazard ratio, 3.6; 95% CI, 2.2–5.9; *p *< 0.001). Despite a better intracranial tumor control rate associated with WBRT, no improvement in survival time occurred. Importantly, fewer patients underwent salvage therapy after SRS plus WBRT than after SRS alone (7.8% vs 32.4%, respectively; difference, − 24.6%; 95% CI, − 35.7 to − 13.5%; *p* < 0.001). In our cohort, as a salvage treatment 21.6% (*n* = 21) of the patients received another CK-SRS, 5.2% (*n* = 5) of the patients needed a surgery and 3.1% (*n* = 3) required a WBRT. These were well-tolerated. In regard of the quality of life, physical and social functioning as well as drowsiness and motor dysfunction deteriorated in our small cohort after SRS with complete questionnaires. However, this subjective evaluation was not reflected in the objective complications. Therefore, we assume that older patients who continue to age during multimodal treatment will experience some decrease in quality of life. However, due to the low number of the questionnaires after the SRS our analysis cannot judge this aspect sufficiently. In the series of Brown et al. [5, 25] there was less cognitive deterioration at 3 months after SRS alone (40/63 patients, 63.5%) than when combined with WBRT (44/48 patients, 91.7%; difference, − 28.2%; 90% CI, − 41.9 to − 14.4%; *p* < 0.001). Quality of life (QoL) was higher at 3 months with SRS alone, including overall quality of life (*p* = 0.001). A retrospective study by Chen et al. [26] compared SRS and WBRT in 119 geriatric patients with overall 811 lesions (≥ 70 years, ≤ 10 brain metastases). In univariate analysis, fatigue, headache and RTOG CNS toxicity (68.0% vs. 89.0%, *p* = 0.009) and KPS decline (2.0% vs. 35%. *p* = 0.0005) was significantly lower in the SRS arm. The multivariate analysis confirmed a higher toxicity after 3 months for the WBRT arm. In contrast to the QUARTZ study, where the combination of WBRT and dexamethasone showed only a small difference in QoL and no difference in OS between the two groups [27], our data with a median survival of 55.6 weeks, preserved KPS and QoL, and no severe acute complications, show a benefit of local therapy with the CK-SRS instead of best supportive care. In light of this accumulated evidence of good local control rates and overall low toxicity rates, SRS should be preferred over WBRT in elderly patients with a certain number of brain metastases despite the limitation for distant tumor control.

As SRS is a purely local therapy, we focused on predictive factors for local control rather than OS and distant progression-free control. The univariate analyses identified older age, female sex, and larger PTV as risk factors for local progression in our cohort, while in the multivariate analysis older age and female sex remained significant, with PTV approximating statistical significance. Importantly, the primary diseases and GPA score did not play a significant role in the regression analysis. We chose the GPA score for our analysis because in two previous studies the score index for radiosurgery (SIR) was superior to the recursive partitioning analysis (RPA) score and in a second analysis the GPA score was the strongest prognostic factor for survival compared to the SIRS [12, 28]. In regard to primary diseases, one possible explanation could be more regular MRI brain scans in some tumor entities such as melanoma in the routine that enables a faster identification of a brain metastases that then response better to a local treatment despite radioresistancy. The prescribed dose did neither play a role in the local control, that is probably due to the overall comparable dose regime in our cohort. Gregucci et al. also identified PTV and BED ≥ 40 Gy as factors associated with local control [18], whereas Minniti et al. could not identify factors predictive of local control [17]. The fact that female sex was identified as a risk factor in our series might be due to the distribution of the primary tumors between the sexes. For instance, breast cancer comprised up to 25% of the primary tumors amongst women and had the worst local control. In this regard, we analyzed the proportion of radioresistant tumors amongst both sexes, however the radiation therapy resistant histologies like malignant melanoma and renal cell carcinoma were balanced between male and female. Triple negative breast cancer was diagnosed in only one woman. One further aspect is the total PTV pro patient that was slightly higher for female patients but did not reach a significant level. Overall, our data did not provide the reason for this observation between female and male patients. We identified older age as a potential risk factor for worse local control; however, Watabene et al. performed a case-matched study comparing SRS applied to very elderly (≥80 years old) patients with that applied to patients between 65 and 79 years old [23] and reported similar local control and complication rates between the groups, signifying that SRS should not be only preserved for patients younger than 80 years [29]. We assume that differences in systemic treatments provided to very elderly patients may explain the effect of age on local control. Although there was no obvious difference in the analysis of the systemic therapy amongst different age groups, we could not supply the exact treatment details. As this was a retrospective study, our data lacked sufficient power to analyze this factor sufficiently.

The major weakness of this study was its retrospective design and the unavoidable preselection of the patients due to the outpatient treatment that requires a reasonable pre-treatment KPS. In any case, our study provides valuable data for this patient group, as it is the largest to date published lesion series concerning elderly patients with brain metastases who were treated using CK.

## Conclusions

Radiosurgical treatment of elderly patients with brain metastases could be a safe and efficient option with preserved KPS and QoL. The multivariate analysis shows older age and female sex were predictive factors of local progression, while for the GPA no significance was detected. This analysis provides a basis for further SRS treatment of elderly patients with a limited number of metastases, despite the GPA classification.

## Data Availability

The datasets generated during and/or analysed during the current study are not publicly available due to the protection of data privacy but are available from the corresponding author on reasonable request as an anonymous set.

## Abbreviations

CK: Cyberknife
CT: Computed tomography
CTCAE: Common terminology criteria for adverse events
GPA: Graded prognostic assessment
GTV: Gross tumor volume
KPS: Karnofsky Performance Score
LINAC: Linear accelerator
MRI: Magnetic resonance images
OS: Overall survival
PD: Prescription dose
PTV: Planning target volume
RPA: Recursive partitioning analysis
SIR: Score index for radiosurgery
SRS: Stereotactic radiosurgery
WBRT: Whole brain radiation therapy
